# Resistance risk asssement and molecular basis of metconazole in *Fusarium pseudograminearum*

**DOI:** 10.1007/s44154-025-00221-0

**Published:** 2025-05-01

**Authors:** Guixiang Li, Yiwen Li, Ling Zhang, Han Jiang, Kang Yuan, Jianqiang Miao, Xili Liu

**Affiliations:** 1 State Key Laboratory for Crop Stress Resistance and High-Efficiency Production, College of Plant Protection, Northwest A&F University, 3 Taicheng Road, Yangling, Shaanxi 712100 China; 2 Department of Plant Pathology, College of Plant Protection, China Agricultural University, 2 Yuanmingyuanxi Road, Beijing, 100193 China

**Keywords:** Metconazole, Resistance mechanism, Resistance risk, *Fusarium pseudograminearum*, Point mutation

## Abstract

The fungicide metconazole, which acts as a sterol 14α-demethylation inhibitor (DMI), can exhibit strong inhibitory effects on *Fusarium pseudograminearum*. However, the resistance mechanism as well as the risk that *F. pseudograminearum* develops resistance to metconazole is yet to be fully assessed. In this study, metconazole displayed a mean EC_50_ value of 0.0559 μg/mL against 105 *F. pseudograminearum* isolates. Ten sensitive parental isolates were then subjected to fungicide adaptation to generate resistant mutants, with in vitro experiments subsequently highlighting the inferior fitness of the mutants. In addition, metconazole exhibited positive cross-resistance with both mefentrifluconazole and tebuconazole. Altogether, the results confirmed the low risk that *F. pseudograminearum* develops resistance to metconazole. Finally, a mutation genotype (M151T) was identified in FpCYP51B, with the mutants also overexpressing the *FpCYP51* genes. Subsequent molecular docking and transformation-based experiments indicated that M151T substitution and overexpression in *FpCYP51* genes conferred resistance to metconazole in *F. pseudograminearum*.

**Supplementary Information:**

The online version contains supplementary material available at 10.1007/s44154-025-00221-0.

## Highlights

The baseline sensitivity of *Fusarium pseudograminearum* to metconazole was established.

*F. pseudograminearum* demonstrated a low resistance risk to metconazole.

The increased expression of the *CYP51* genes as well as M151T mutations in FpCYP51B could confer metconazole resistance.

**Supplementary Information:**

The online version contains supplementary material available at 10.1007/s44154-025-00221-0.

## Introduction

Fusarium crown rot (FCR), a highly damaging soil-borne disease that is primarily caused by *Fusarium pseudograminearum*, affects a number of cereal crops, including wheat (Kazan and Gardiner 2018). Recent years have witnessed a surge in the prevalence of this disease, resulting in significant losses in many wheat-producing countries (Smiley et al. 2005). For instance, in Australia, FCR can regularly cause losses of up to 10% in cereal crops. Similarly, during seasons that particularly favor the disease, losses may range from 8 to 36% for bread wheat and from 24 to 52% for durum wheat (Hollaway et al. 2013). In China, FCR was initially reported in 2011 (Li et al. 2012), and since then, it has been identified in many provinces, including Hebei, Henan, Shandong, Shaanxi, Jiangsu and Anhui (Zhou et al. 2019; Fan et al. 2021). According to estimates, the disease typically results in a 10% yield loss in wheat, with this value even rising to over 30% under conditions that favor FCR (Zhang et al. 2020a). Furthermore, recent studies stated that between 2017 and 2021 alone, FCR decreased the average yearly yield by 36,000 tons in the Henan Province, with a peak annual reduction of 44,000 tons (Zhao et al. 2022). The main symptoms of FCR include browning and rot at the base of stems, whitening or pinking of the internodal sections as well as whiteheads at the harvesting stage (Kazan and Gardiner 2018). In addition, there have been reports regarding *F. pseudograminearum*’s ability to produce various mycotoxins, including zearalenone, 15-acetyl-deoxynivalenol, 3-acetyl-deoxynivalenol and deoxynivalenol that may negatively impact human and animal health (Fan et al. 2021; Obanor et al. 2012). So far, FCR is mainly managed by fungicide application due to changes in tillage practices as well as the lack of effective resistant varieties (Alahmad et al. 2018). However, as far as China is concerned, only few fungicides have been approved for use in controlling the disease, and these include 200 g/L cyclobutrifluram flowable concentrate for seed treatment (FS), 33% fludioxonil**·**clothianidin flowable concentrate for seed coating (FSC) and 40% prothioconazole**·**tebuconazole suspension concentrate (SC) (www.chinapesticide.org.cn). Hence, it is crucial that new and effective fungicides are introduced for the successful control of FCR. The fungicide metconazole, classified as a 14α-demethylation inhibitor (DMI) with FRAC Code 3, was developed by the Kureha Corporation in 1986 (Kumazawa et al. 2000). First registered in France in 1993 as a fungicide for cereals, it was then applied for the management of several diseases, such as leaf rust, Septoria blotch and powdery mildew (Spolti et al. 2012). When metconazole comes into contact with the targeted fungi, it disrupts ergosterol synthesis by inhibiting CYP51’s activity which is responsible for catalyzing the demethylation at C14 of lanosterol or 24-methylene dihydrolanosterol. As a result, the function of fungal cell membranes is impaired (Liu et al. 2011; Wei et al. 2023). Interestingly, Duan et al. found that metconazole demonstrated significant fungicidal activity against the mycelial growth of Chinese Fusarium head blight pathogens (Duan et al. 2018).

A previous study also reported the significant antifungal effects of metconazole against *F. pseudograminearum* strains obtained from key wheat-producing regions in China (Liu et al. 2023). However, before registering a fungicide for disease management, it is essential to evaluate the risk that a pathogen becomes resistant to it (Brent and Hollomon 2007). In this context, DMIs are classified as having a low to medium risk of inducing resistance, as assessed by the Fungicide Resistance Action Committee (https://www.frac.info/). Nevertheless, there are still uncertainties regarding *F. pseudograminearum*’s potential in developing resistance to metconazole. Thus, the objectives of study were to: (i) establish the baseline sensitivity of *F. pseudograminearum*, isolated from Chinese provinces, to metconazole, (ii) analyze the likelihood that the isolates develop resistance to the fungicide and (iii) investigate the underlying mechanism contributing to *F. pseudograminearum*’s resistance to metconazole.

## Results

### *F. pseudograminearum*’s baseline sensitivity to metconazole

**Fig. 1:**
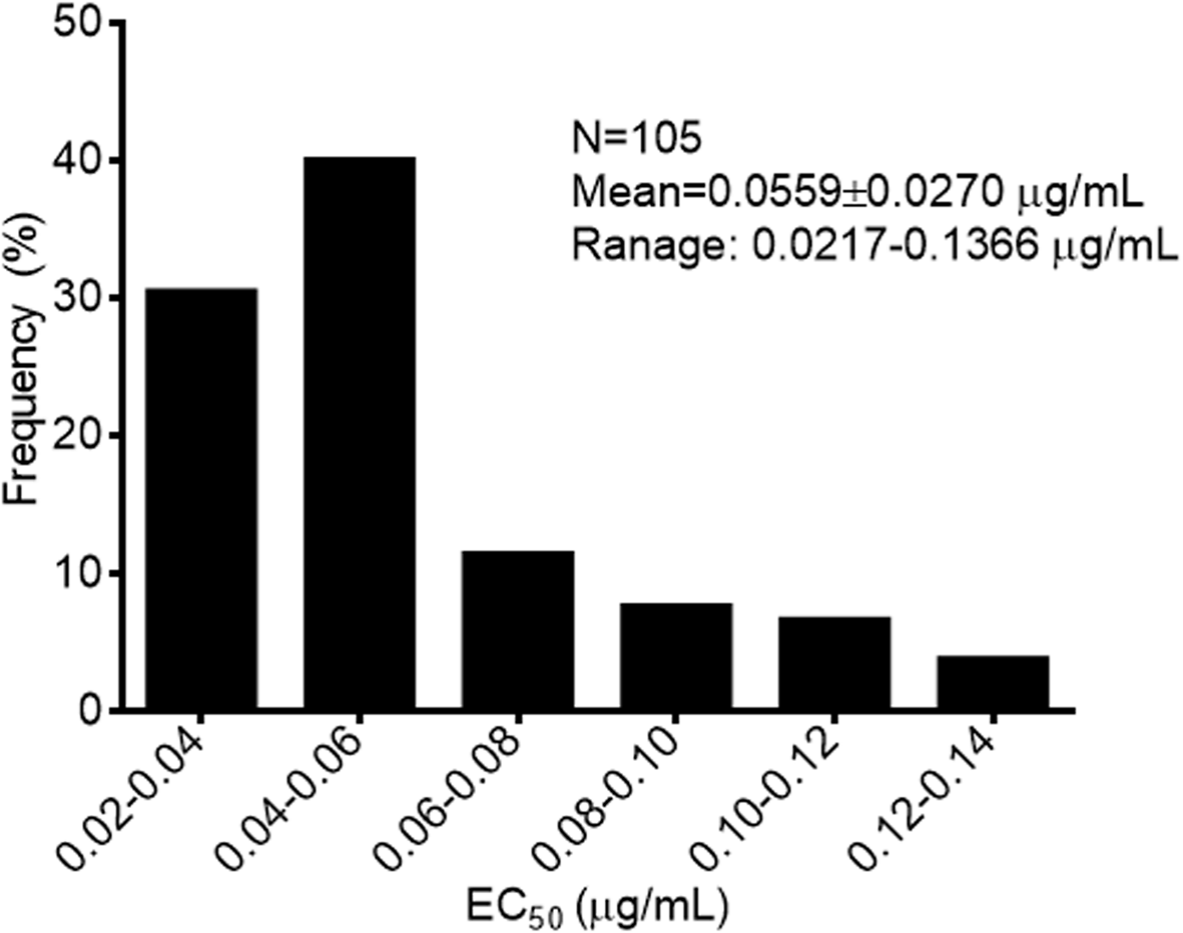
Frequency distribution of metconazole EC_50_ values for 105 *Fusarium pseudograminearum* isolates

The 105 *F. pseudograminearum* isolates exposed to metconazole had EC_50_ values that varied between 0.0217 and 0.1366 μg/mL. A unimodal pattern of distribution, with a mean EC_50_ value of 0.0559 μg/mL, was also observed (Fig. 1), and hence, no metconazole-resistant subpopulations were present among the strains under study. In addition, even though the *F. pseudograminearum* isolates were obtained from four distinct geographical regions, they did not exhibit variations in their sensitivity to metconazole (Table 1).

**Table 1:** Sensitivity of 105 *Fusarium pseudograminearum* isolates collected from four provinces in China to metconazole

province	number of isolates	EC_50_ (µg/mL)	
		**range**	**mean**^***a***^
Shandong	29	0.023–0.131	0.058 a
Shaanxi	44	0.030–0.137	0.057 a
Henan	14	0.024–0.094	0.047 a
Hebei	18	0.022–0.129	0.057 a

^*a*^Values followed by the same letters with a column are not significantly different (*P* < 0.05)

### Stability of metconazole-resistant mutants

Three sensitive isolates (FP4, W6, and H11) were used to generate six metconazole-resistant mutants (FP4-7–1, FP4-7–2, W6-3, W6-4, H11-5–1 and H11-5–2) through fungicide adaption. In this case, the mutants’ RFs varied between 11.09 and 97.72, but after repeated subculturing on fungicide-free PDA (potato dextrose agar) 10 times, the RFs of the 10th generation ranged from 12.40 to 51.88 (Table 2). The metconazole-resistance was relatively stable in three mutants (FP4-7–2, W6-3, W6-4), with little change in the resistance factor; however, in the other three mutants (FP4-7–1, H11-5–1, H11-5–1), the resistance factor slightly decreased, indicating that the metconazole-resistance was unstable in these three mutants (Table 2).

**Table 2:** Metconazole-reisitance stability six *Fusarium pseudograminearum* mutants and their parental isolates after the first and tenth subculture on fungicide-free medium

Isolates^*a*^	origin	EC_50_ (µg/mL)		RF^*b*^		FSC^*c*^
		**first**	**tenth**	**first**	**tenth**	
FP4	parent	0.0256	0.0358	-	-	-
FP4-7–1	mutant	1.0932	1.0273	42.70	28.69	0.67
FP4-7–2	mutant	0.9648	1.6093	37.69	44.95	1.19
W6	parent	0.0346	0.0343	-	-	-
W6-3	mutant	0.3827	0.4253	11.09	12.40	1.12
W6-4	mutant	0.6342	1.0694	18.38	31.18	1.70
H11	parent	0.0170	0.0585	-	-	-
H11-5–1	mutant	1.3185	1.3164	77.56	22.50	0.29
H11-5–2	mutant	1.6612	3.0354	97.72	51.88	0.53

^*a*^FP4, W6 and H11 are the wide-type parental isolates; ^*b*^RF, resistance factor; ratio of EC_50_ of resistant mutant and EC_50_ of sensitive parental isolate; ^*c*^*FSC* factor of sensitivity change; ratio of RF of the tenth transfer to that of the first transfer on fungicide-free medium

### Effects of varying temperatures on fungal growth

A temperature of 25 °C was found to be optimum for the growth of both the sensitive and metconazole-resistant strains. However, growth was entirely suppressed at 37 °C. The mutants H11-5–1 and H11-5–2 had comparable growth rates in comparison to their parental strains at temperatures of 25 °C and 30 °C. Interestingly, mutants FP4-7–1, FP4-7–2, W6-3 and W6-4 exhibited lower growth rates compared with their corresponding parental isolates at temperatures of 4 °C, 13 °C, 18 °C, 25 °C and 30 °C (Table 3).

**Table 3:** Mycelial growth of metconazole-resistant *Fusarium pseudograminearum* mutants and their parental isolates on PDA plates at various temperatures

isolates	colony diameter (cm)^*a*^					
	**4 ℃**	**13 ℃**	**18 ℃**	**25 ℃**	**30 ℃**	**37 ℃**
FP4	3.13 ± 0.05 a	4.35 ± 0.09 a	6.43 ± 0.09 a	7.54 ± 0.10 a	6.48 ± 0.18 a	-
FP4-7–1	1.77 ± 0.03 b	1.99 ± 0.09 b	3.36 ± 0.16 b	3.97 ± 0.09 b	3.62 ± 0.08 b	-
FP4-7–2	1.51 ± 0.08 c	2.04 ± 0.02 b	3.28 ± 0.11 b	3.94 ± 0.08 b	3.67 ± 0.03 b	-
W6	2.52 ± 0.06 a	3.96 ± 0.11 a	6.08 ± 0.12 a	7.52 ± 0.06 a	6.65 ± 0.01 a	-
W6-3	1.66 ± 0.02 c	2.14 ± 0.03 c	3.87 ± 0.07 c	5.10 ± 0.03 c	4.64 ± 0.01 b	-
W6-4	2.07 ± 0.01 b	2.35 ± 0.02 b	4.98 ± 0.02 b	5.40 ± 0.09 b	3.66 ± 0.18 c	-
H11	2.97 ± 0.10 a	3.04 ± 0.01 a	4.71 ± 0.05 a	4.99 ± 0.02 a	4.17 ± 0.01 a	-
H11-5–1	1.81 ± 0.03 c	2.19 ± 0.03 c	4.03 ± 0.06 b	4.83 ± 0.07 a	4.20 ± 0.07 a	-
H11-5–2	2.15 ± 0.01 b	2.39 ± 0.02 b	3.48 ± 0.03 c	4.87 ± 0.06 a	4.26 ± 0.01 a	-

^*a*^Colony diameters were measured 3 days post inculation. Values followed by the same letter within a column do not differ significantly (*P* < 0.05); Note: ‘-’ indicates that the strain does not grow

### Fitness studies of parental and metconazole-resistant *F. pseudograminearum* isolates

**Fig. 2:**
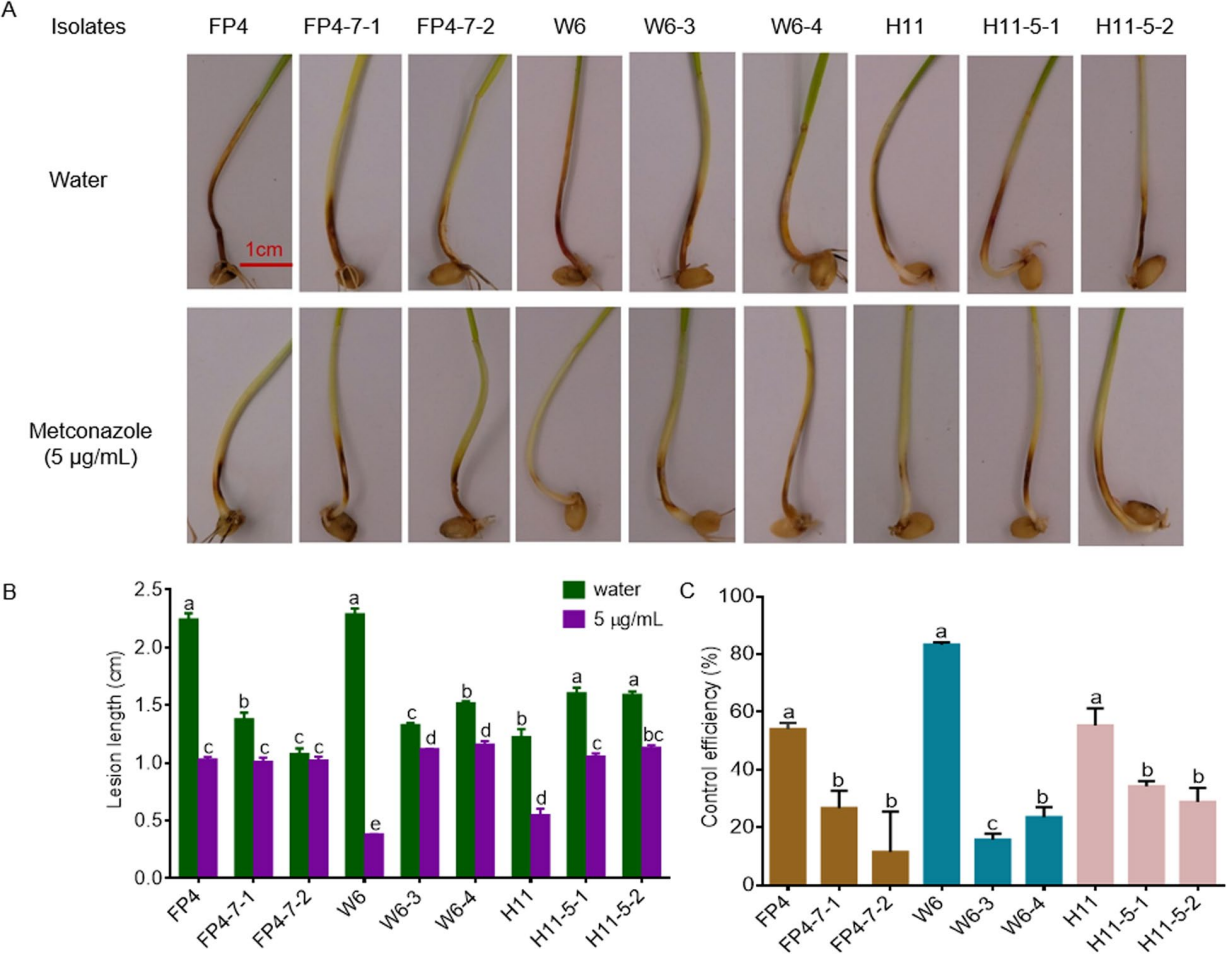
Control efficacy of metconazole to *Fusarium pseudograminearum*. **A** Representative images of wheat coleoptiles 4 days post-inoculation with the *Fusarium pseudograminearum* isolates. **B** The lesion length on wheat coleoptiles sprayed with water (control) or 5 μg/mL metconazole treatment were inoculated with mycelial plugs of each *F*. *pseudograminearum* isolate at 4 days post-inoculation. **C** Control efficacy of metconazole on *F*. *pseudograminearum* resistant mutants and parental isolates

Conidia production was determined. Overall, even though the six mutants exhibited significantly lower conidiation, their conidial germination rates were still comparable to that of their respective parental isolates. Furthermore, compared with the metconazole-sensitive strains, only two mutants (H11-5–1 and H11-5–2) were found to be more pathogenic, with the other four (FP4-7–1, FP4-7–2, W6-3 and W6-4) displaying significantly lower pathogenicity (Fig. 2A and B). The mutants’ fitness was also assessed based on a composite index, and as shown in Table 4, their competitive fitness index (CFI) values were notably lower in comparison with the corresponding parental isolates. These results suggested that the metconazole-resistant mutants possess a fitness penalty.

**Table 4:** Biological characteristics of metconazole-resistant *Fusarium pseudograminearum* and the wild-type sensitive parental isolates^*a*^

isolate	colony diameter(cm)	conidiation (× 10^5^/mL)	germination percentage of conidium(%)	lesion length^*b*^ (cm)	CFI^*c*^ (× 10^8^)
FP4	7.54 ± 0.10 a	12.82 ± 0.56 a	89.33 ± 1.33 a	2.24 ± 0.18 a	19.34 a
FP4-7–1	3.97 ± 0.09 b	10.53 ± 0.57 b	89.67 ± 2.33 a	1.37 ± 0.06 b	5.14 b
FP4-7–2	3.94 ± 0.08 b	10.57 ± 0.61 b	91.00 ± 2.08 a	1.08 ± 0.03 c	4.09 c
W6	7.52 ± 0.06 a	10.91 ± 0.94 a	98.67 ± 0.88 a	2.28 ± 0.04 a	18.46 a
W6-3	5.10 ± 0.03 c	5.46 ± 0.66 b	98.67 ± 0.33 a	1.32 ± 0.09 c	3.63 c
W6-4	5.40 ± 0.09 b	6.48 ± 0.37 b	98.33 ± 0.88 a	1.52 ± 0.06 b	5.23 b
H11	4.99 ± 0.02 a	10.00 ± 0.15 a	97.67 ± 1.33 a	1.21 ± 0.08 b	5.90 a
H11-5–1	4.83 ± 0.07 a	4.97 ± 0.50 b	96.33 ± 0.67 a	1.60 ± 0.06 a	3.70 b
H11-5–2	4.87 ± 0.06 a	3.92 ± 0.65 b	94.67 ± 0.88 a	1.59 ± 0.05 a	2.87 c

^*a*^Values followed by the different letters within a column represent significant difference between mutants and their corresponding parental isolates (*P* < 0.05); ^*b*^Length of lesions on a susceptible wheat cultivar (Xiaoyan 22) inoculated with mycelia plug was measured at 4 dpi;^*c*^Compound fitness index (CFI) = mycelial growth at 25°C × conidiation × conidial germination × lesion length

### Efficacy of metconazole against mutants on wheat coleoptile

Metconazole was more effective against parental isolates compared with mutants. Indeed, applying metconazole to wheat coleoptiles that had been infected with the mutants resulted in significantly longer lesions as opposed to those observed after infection with the sensitive strains (Fig. 2A and B). Specifically, parental isolates were well controlled at 5 μg/mL metconazole, with the efficacy being 54.08%, 83.38% and 55.28% for the sensitive isolates FP4, W6 and H11, respectively. In contrast, at a similar concentration, the mutants were not well controlled, with the efficacy of the resistant isolates FP4-7–1, FP4-7–2, W6-3, W6-4, H11-5–1 and H11-5–2 being 26.66%, 11.49%, 15.74%, 23.55%, 34.26% and 28.83%, respectively (Fig. 2C).

### Cross-resistance

**Fig. 3:**
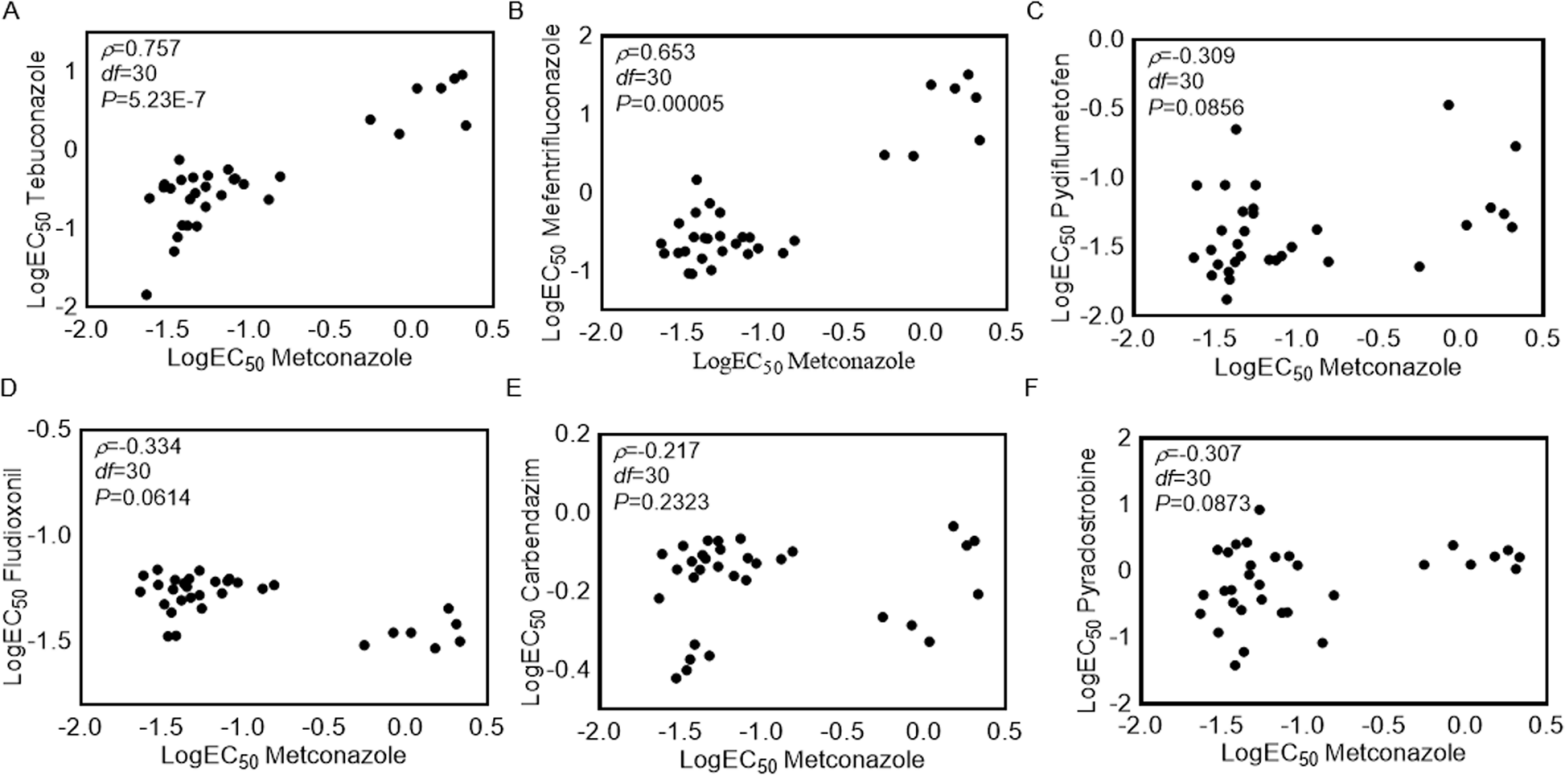
Spearman’s rank correlation coefficients for cross-resistance between metconazole and other fungicides. **A** tebuconazole; (**B**) mefentrifluconazole; (**C**) pydiflumetofen; (**D**) fludioxonil; (**E**) carbendazim; (**F**) pyraclostrobin

Metconazole exhibited a positive cross-resistance with both tebuconazole and mefentrifluconazole (*p* < 0.05, Fig. 3A and B). However, no cross-resistance was observed between metconazole and the other four fungicides (pyraclostrobin, fludioxonil, carbendazim, and pydiflumetofen) (*p* > 0.05, Fig. 3C-F).

### Comparing the *FpCYP51* sequences of mutant and parental strains

**Fig. 4:**
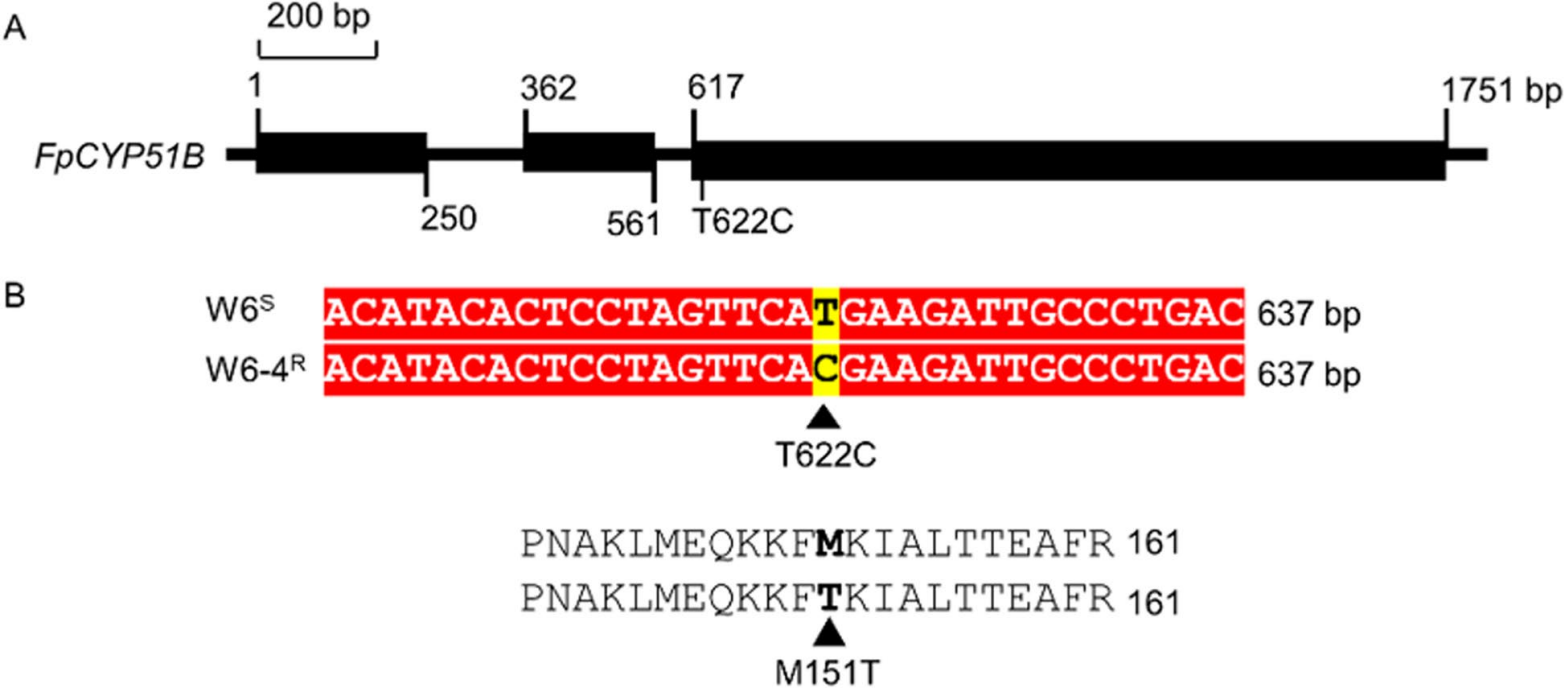
Multiple sequence alignment of the CYP51B protein from metconazole-resistant *Fusarium pseudograminearum* mutants and their corresponding parental isolates. **A** Schematic representation of *F. pseudograminearum FpCYP51B*. **B** Alignment of partial deduced amino acid sequence of FpCYP51B from metconazole-sensitive parental strains W6 and metconazole-resistant mutant W6-4. Exonic regions of genes are indicated with black rectangle and introns are indicated with black line in (A)

DNA sequence alignment revealed the absence of point mutations in the *FpCYP51A* and *FpCYP51C* genes of the six metconazole-resistant mutants. However, the *FpCYP51B* gene of mutant W6-4 possessed a point mutation ATG → ACG that led to the M151T change (Fig. 4 and S2).

### Effects of FpCYP51B-M151T substitution on metconazole’s sensitivity

**Fig. 5:**
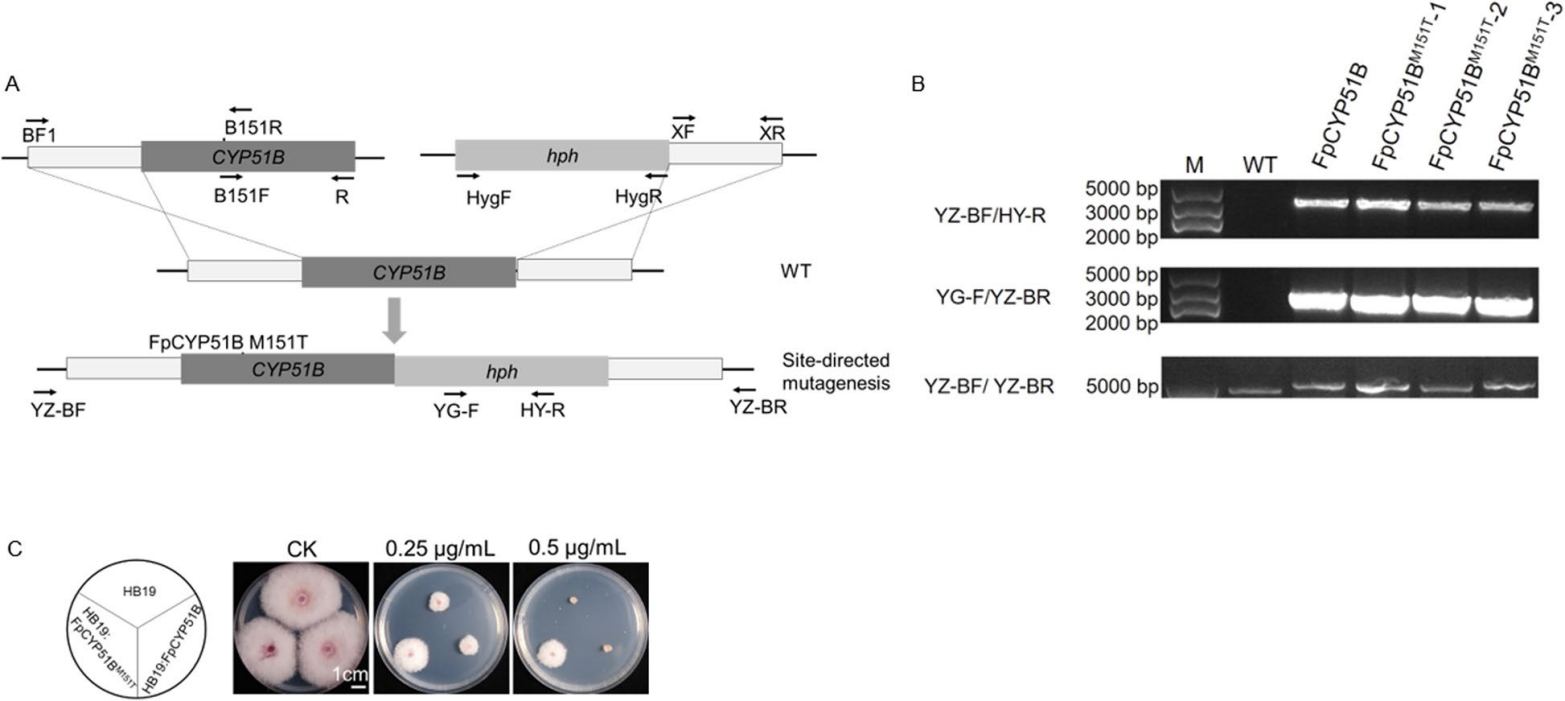
Growth and PCR validation of *Fusarium pseudograminearum* transformants. **A** Schematic diagram of FpCYP51B homologous recombination. **B** Electrophoresis gel image of PCR products for transformants validation. The primer pair YZ-BF/HY-R was used to specifically amplify upstream-HPH fragment (3480 bp), primer pairs YG-F/YZ-BR was used to specifically amplify HPH-downstream fragment (2770 bp), and primer pairs YZ-BF/YZ-BR were used to amplify upstream-HPH-downstream fragment (5935 bp) for site-directed mutants. **C** Growth of *F*. *pseudograminearum* transformants on PDA plates amended with 0.25 µg/mL and 0.5 µg/mL metconazole

Through protoplast transformation mediated by homologous recombination, we successfully obtained five transformants, and these included two controls having the wild-type allele (HB19-FpCYP51B-1/2) as well as three transformants that carried the M151T mutation (HB19-FpCYP51B^M151T^-1/2/3). The sensitivity of all strains to metconazole was then tested, with all successful transformations confirmed by PCR (Fig. 5A-B). Overall, the HB19-FpCYP51B^M151T^-1/2/3 substitution strains exhibited lower sensitivity to metconazole compared with the wild-type HB19, as reflected in their EC_50_ values. On the other hand, there was no change in the sensitivity of the two control transformants to metconazole (Fig. 5C and Table 5). The findings confirmed that *F. pseudograminearum*’s sensitivity to metconazole was reduced as a result of the M151T point mutation in FpCYP51B.

**Table 5:** The EC_50_ to metconazole and resistance factor of *Fusarium pseudograminearum* transformants

isolate	EC_50_ (µg/mL)^*a*^	95% Confidence interval	RF value^*b*^
HB19	0.0421	0.0315–0.0563	-
HB19-FpCYP51-1	0.0449	0.0204–0.0987	-
HB19-FpCYP51-2	0.0533	0.0221–0.1288	-
HB19-FpCYP51^M151T^-1	0.1492	0.0924–0.2409	3.55
HB19-FpCYP51^M151T^-2	0.1308	0.0902–0.1897	3.29
HB19-FpCYP51^M151T^-3	0.1344	0.1130–0.1598	3.19

^*a*^EC_50_ to metconazole of transformants with point mutation or not was determined using a mycelial inhibition method; ^*b*^RF value, ratio of EC_50_ of the transformants with point mutation to the average of EC_50_ of that without point mutation

### Molecular docking

**Fig. 6:**
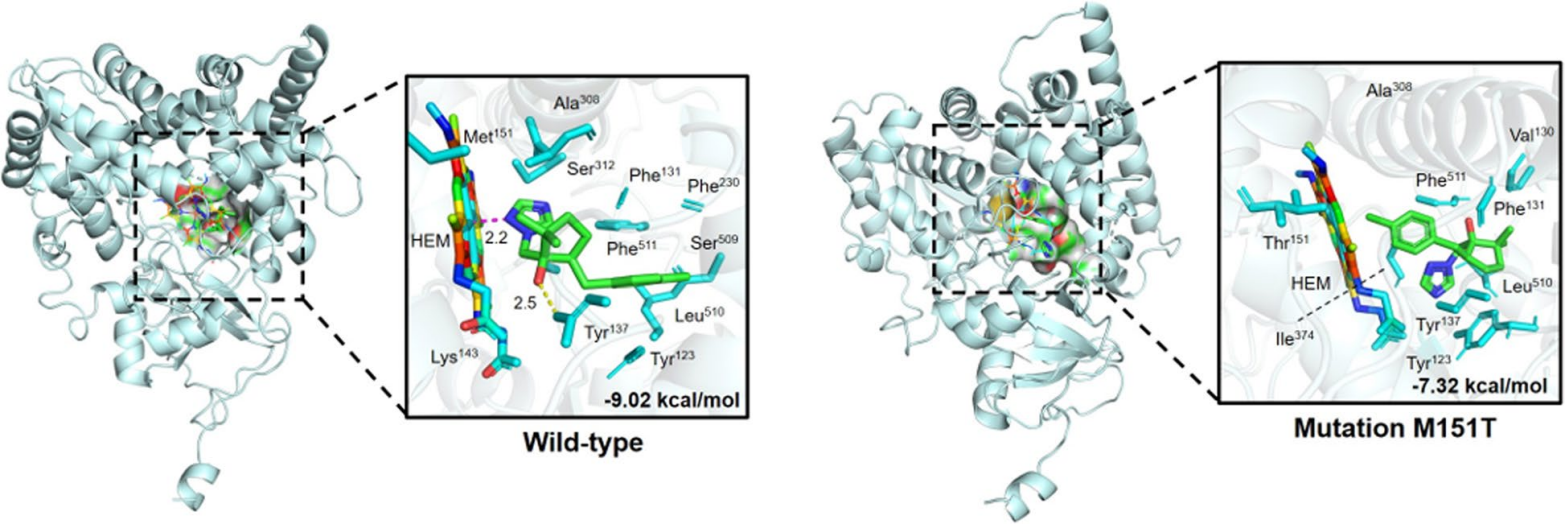
Docking of metconazole with FpCYP51B proteins from resistant mutants and sensitive parental isolates

Analyses based on molecular docking indicated that the M151T mutation in FpCYP51B altered the agent’s mode of action on target proteins, with its binding energy to metconazole also reduced (Fig. 6). This particular mutation, which prevents the formation of a hydrogen bond at position 137, has been linked to azole resistance (Qian et al. 2018), and this was reflected in the change in binding affinity scores which decreased from -9.02 to -7.32 kcal/mol. Altogether, the findings provided evidence that the M151T mutation reduced *F. pseudograminearum*’s affinity to metconazole.

### Expression levels of* FpCYP51* genes and sensitivity of* FpCYP51A/B*-overexpressing transformants to metconazole

**Fig. 7:**
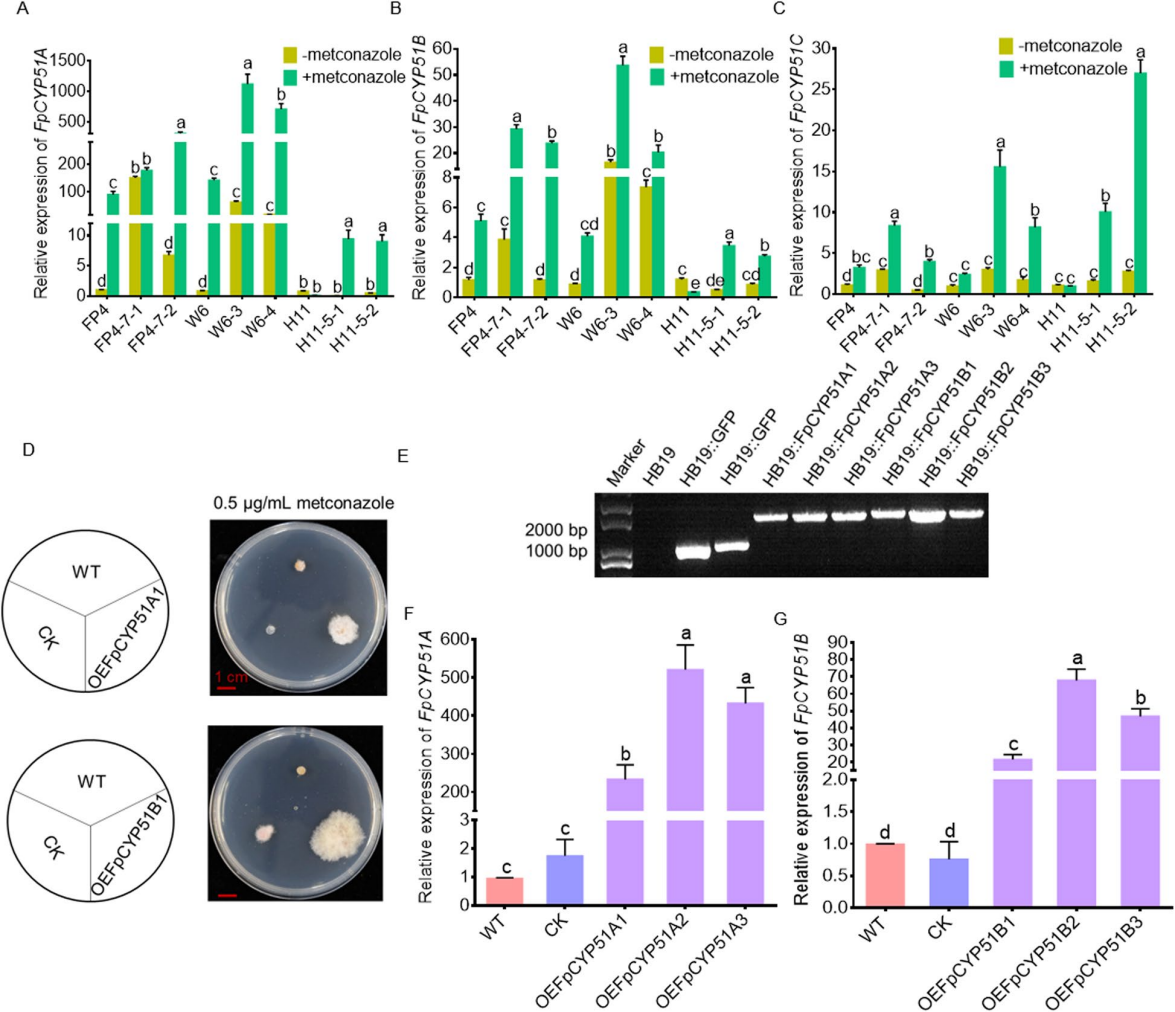
Expression level of *FpCYP51s* and growth, PCR validation, and *FpCYP51A/B* expression level of overexpression transformants. **A** Expression level of *FpCYP51A,* (**B**) *FpCYP51B* and (**C**) *FpCYP51C* in metconazole-sensitive *Fusarium pseudograminearum* strains FP4, W6, H11 and metconazole-resistant mutants FP4-7–1, FP4-7–2, W6-3, W6-4, H11-5–1, and H11-5–2. **D** Growth of *F. pseudograminearum* transformants on PDA plates amended with 0.5 μg/mL metconazole for 7 d. **E** Electrophoresis gel image of PCR products for transformants validation. **F** *FpCYP51A* and (**G**) *FpCYP51B* expression level in related transformants

Quantitative RT-PCR revealed increased expression of *FpCYP51A* and *FpCYP51B* in the FP4-7–1, W6-3 and W6-4 mutants (Fig. 7A and B), with the *FpCYP51C* gene also upregulated in FP4-7–1 when metconazole was absent (Fig. 7C). However, when metconazole was present, the resistant strains exhibited a marked increase in the expression of *FpCYP51* genes compared with the sensitive ones, except in the case of the *FpCYP51C* gene in FP4-7–2. The results suggested that resistance to metconazole could be attributed to changes in the relative expression level of *FpCYP51* genes. Further experiments of overexpression demonstrated that the sensitivity of *FpCYP51A*-overexpressing transformants to metconazole decreased (Fig. 7D-G), with RF values ranging from 1.61 to 1.84, while the sensitivity of *FpCYP51B*-overexpressing transformants to metconazole also decreased, with RF values ranging from 4.65 to 8.51 (Table 6).

**Table 6:** Sensitivity of *Fusarium pseudograminearum* to metconazole

isolate	EC_50_ (µg/mL)^*a*^	95% Confidence interval	RF value^*b*^
HB19	0.017	0.008–0.039	-
CK	0.010	0.004–0.029	0.60
OEFpCYP51A1	0.028	0.010–0.078	1.61
OEFpCYP51A2	0.032	0.020–0.049	1.82
OEFpCYP51A3	0.032	0.014–0.074	1.84
OEFpCYP51B1	0.148	0.056–0.392	8.51
OEFpCYP51B2	0.081	0.053–0.125	4.65
OEFpCYP51B3	0.113	0.073–0.175	6.49

^*a*^EC_50_ to metconazole of over-expressed transformants was determined using a mycelial inhibition method; ^*b*^RF (resistant factor) value, ratio of EC_50_ of over-expressed transformants to the EC_50_ of that wild-type parental isolate

## Discussion

FCR, a major disease primarily caused by *Fusarium* species, restricts crop yield in the Huanghuai wheat-growing area of China (Deng et al. 2019). This disease can cause substantial losses, and as such, it poses a serious threat to wheat cultivation. More recently, there has been a surge in the prevalence of FCR due to several factors, including the adoption of minimum tillage farming practices, the accumulation of pathogenic fungal sources as well as the lack of resistant varieties (Burgess et al. 1996). Consequently, both the prevalence and severity of the disease have increased, thereby significantly challenging the production and quality of wheat (Smiley et al. 2005; Deng et al. 2019). From a practical perspective, the application of biological approaches and good agricultural practices for disease control have certain limitations (Wei et al. 2023), and this resulted in the wide adoption of chemical methods which are now recognized as an indispensable approach for disease management due to their quick and efficient characteristics (Zhou et al. 2024). However, in China, the number of fungicides currently approved for managing FCR is relatively limited. This restricts farmers’ ability to implement appropriate chemical control measures when facing diseases. Therefore, accelerating the registration and approval of more fungicides is crucial to provide farmers with a wider range of effective control options.

However, in China, additional research that examines the risk of fungicide resistance is required before registering a particular fungicide. In this context, the baseline sensitivity is crucial for assessing the risk that pathogen populations develop resistance and for monitoring resistance levels (Miao et al. 2016). This study assessed the sensitivity of 105 *F. pseudograminearum* isolates to metconazole, and the results revealed an average EC_50_ value of 0.0559 ± 0.0270 μg/mL. The sensitivity distribution also exhibited a nearly unimodal shape, thereby suggesting that resistant subpopulations were absent from the field population. These results highlight the efficacy of metconazole against the isolates tested while providing critical insights for monitoring changes in *F. pseudograminearum*’s sensitivity to metconazole.

Studies on fitness penalties are essential for evaluating the risk of resistance (Hawkins and Fraaije 2018). This work found that resistant mutants exhibited lower fitness compared with their parent isolates. This reduction in fitness could have implications for the persistence and spread of resistance within populations. For instance, Wei et al*.* reported that prothioconazole-resistant mutants could exhibit varying levels of deficiencies in pathogenicity, conidia production and vegetative growth on wheat. They speculated that the trade-off between prothioconazole resistance and biological fitness in mutants could result in their inability to compete with *F. pseudograminearum*-sensitive populations in the field (Wei et al. 2023). Altogether, the study’s findings indicated a low risk that *F. pseudograminearum* develops resistance to metconazole. Subsequent assays further demonstrated the absence of significant cross-resistance for metconazole with pydiflumetofen, fludioxonil, carbendazim and pyraclostrobin. Therefore, metconazole could represent an effective option for managing this disease, with the results also highlighting the potential of applying these fungicides alternately or as a mixture with metconazole.

A number of studies have focused on the mechanisms of DMI resistance in fungi, with the most widely reported one being point mutations in CYP51 (Li et al. 2024a, b; Li et al. 2023a, b ; Lichtemberg et al. 2017). For example, the FgCYP51A protein of metconazole-resistant mutants were found to harbor three mutation genotypes: a combined mutation (E103Q and V157L) as well as two single ones (G443S and D243N) (Duan et al. 2018). Similarly, *Fusarium graminearum* were found to exhibit lower sensitivity to tebuconazole as a result of a Y137H mutation in the cytochrome P450 FgCYP51B protein (Qian et al. 2018), while Y123H point mutations in FgCYP51B were shown to induce prochloraz-resistant mutants (Zhao et al. 2021). In the present study, a metconazole-resistant mutant (W6-4 mutant) harbored the M151T substitution in the CYP51B protein, but none of the mutants exhibited altered promoter regions or changes in the *FpCYP51A* and *FpCYP51C* genes. Subsequent alignment of the *F. pseudograminearum*’s CYP51B indicated that the M151T mutation was homologous to I145F in *Phakopsora pachyrhizi*. It is known that I145F induces a loss of sensitivity to DMIs (Schmitz et al. 2013). Moreover, molecular docking and transformation experiments further confirmed the connection between the point mutation in FpCYP51B and metconazole resistance in *F. pseudograminearum*. The binding affinity of FpCYP51B to metconazole was also reduced by the M151T site-directed mutation, with the transformants subsequently displaying lower sensitivity. Finally, the RF values ranged from 3.19 to 3.55 and were different from those of the metconazole-resistant mutants. These observations could be attributed to the overexpression of *FpCYP51s* which contributed to metconazole resistance in *F. pseudograminearum*.

Beside mutations within the *CYP51* gene, there have been reports of other mechanisms through which sensitivity to DMIs is altered, with one example being the insertion of promoter regions that leads to overexpression of the *CYP51* gene (Wei et al. 2023; Li et al. 2023a, b). Similarly, in *F. fujikuroi*, higher expression of *FfCYP51A* and *FfCYP51B* induced a prochloraz-resistant phenotype (Zhang et al. 2020b), while in the case of *Fusarium oxysporum* f. sp. *Niveum*, analysis of *CYP51A* expression revealed a notable increase in the highly prothioconazole-resistant mutants compared with the sensitive controls (Hudson et al. 2021). In this study, the resistant mutants exhibited significantly higher expression levels of *CYP51* genes, but no changes were noted in their promoter regions as revealed by sequence analysis. Moreover, transformation experiments provided additional confirmation of the association between the overexpression of *FpCYP51A or FpCYP51B* and metconazole resistance in *F. pseudograminearum*. Furthermore, transcription factors are also known to be significantly involved in DMI resistance. For instance, in response to tebuconazole treatment, the phosphorylated FgSR modulates the transcription of sterol biosynthesis genes through the SWI/SNF complex (Liu et al. 2019). Therefore, additional investigations are required to fully uncover the mechanisms underlying altered *CYP51* expression in *F. pseudograminearum*.

## Conclusions

This work highlights the low risk that *F. pseudograminearum* develops resistance to metconazole. The observed resistance to metconazole could very likely be attributed to the increased expression of the *CYP51* genes as well as M151T mutations in FpCYP51B. Furthermore, this research provides valuable insights for the monitoring of metconazole resistance and the development of management strategies against *F*. *pseudograminearum*.

## Materials and methods

### Preparation of medium and fungicide treatment

For the routine growth and preservation of *F. pseudograminearum* isolates, potato dextrose agar (PDA) and potato dextrose broth (PDB) were used. These two media, consisting of 20 g/L of dextrose, 200 g/L of potato and 15 g/L of agar (for PDA only), were also used for assessing the fitness of the isolates as well as their sensitivity to fungicides. In addition, *F. pseudograminearum*’s sensitivity to pydiflumetofen was tested using yeast peptone acetate (YBA) medium (10 g/L of yeast extract, 20 g/L of sodium acetate, 10 g/L of bacto peptone and 15 g/L of agar), while mung bean broth (MBB) (containing 30 g/L of mung bean) as well as carboxymethyl cellulose (CMC) liquid medium (1 g of NH_4_NO_3_, 0.5 g of MgSO_4_·7H_2_O, 1 g of KCl, 1 g of KH_2_PO_4_, 15 g/L of sodium carboxymethyl cellulose and 1 g/L of yeast extract) were used to promote the formation of conidia in the fungal isolates (Wang et al. 2024). PDA, PDB and MBB were prepared in distilled water, while for the preparation of YBA and CMC, deionized water was used.

*F. pseudograminearum* isolates were treated with metconazole (95.0% active ingredient, a.i.), supplied by Adama Huifeng Co., Ltd. (Jiangsu), to test their baseline sensitivity and generate resistant mutants. The other fungicides, namely fludioxonil (97% a.i.) and pydiflumetofen (99.7% a.i.) were obtained from Syngenta Investment Co., Ltd., while pyraclostrobin (97% a.i.) as well as mefentrifluconazole (98.8% a.i.) were purchased from BASF Co., Ltd. (Shanghai). Finally, carbendazim (98% a.i.) and tebuconazole (96% a.i.) were obtained from Anhui Guangxin Agrochemical Co., Ltd. (Anhui) and Bayer Crop Science (Shanghai), respectively. All fungicides were of technical grade, and stock solutions of 10^4^ μg/mL were prepared in dimethyl sulfoxide (DMSO), except for carbendazim where a stock solution of 0.1 mol/L was prepared in hydrochloric acid. The stock solutions were subsequently kept at 4℃ until required for use.

### Isolation of* F. pseudograminearum*

This study involved 105 isolates obtained from infected wheat collected across the Chinese provinces of Hebei, Shandong, Henan and Shaanxi in 2020. All the isolates were identified based on their morphological characteristics prior to PCR-based identification. In the latter case, the Fp1–1/Fp1–2 primer pair was used for PCR (Aoki and O'Donnell 1999), with additional information regarding the primers provided in Table S1. All fungal cultures were maintained on PDA medium in plastic tubes and kept at 4 °C in the dark to ensure extended storage.

### *F. pseudograminearum*’s baseline sensitivity to metconazole

The inhibitory effects of metconazole on mycelial growth was measured to assess *F. pseudograminearum*’s sensitivity to the fungicide (Duan et al. 2018). Fresh plugs (5 mm in diameter) were collected from the advancing edge of the mycelia and transferred onto PDA plates containing 0, 0.001, 0.005, 0.01, 0.05, 0.1 or 0.5 μg/mL of metconazole. For the control, 0.1% of DMSO solution was added to the medium. The plates were then incubated in the dark for five days at 25 °C before measuring the colonies. The median effective concentrations EC_50_ value was calculated according to a previously described formula (Li et al. 2023a, b, c; Fei et al. 2023). Each treatment involved three replicates.

### Generating metconazole-resistant mutants from wild-type *F. pseudograminearum*

To determine the likelihood that *F. pseudograminearum* develops resistance to metconazole, ten wild-type isolates obtained from various regions (DC3, HB1, H11, DE2, W6, DE13, HN-0, DA17, FP4 and HB19) were cultured on PDA before being incubated for five days at 25 °C. Fresh 5-mm wide mycelial plugs were then collected from the relevant sensitive strains and kept on PDA plates to which metconazole had been added (final concentration of 0.5 μg/mL). Following 15 to 20 days of incubation in the dark at 25 °C, fresh PDA medium having a similar concentration of metconazole was overlaid onto plates showing hyphal growth. Sectors showing rapid growth were subsequently moved to fungicide-free PDA plates and after a five-day incubation, mycelial plugs were transferred to plates with gradually increasing concentrations of metconazole (1, 2.5, 5, 10, 20 and 30 μg/mL). The above selection procedure was performed until normal growth was observed on PDA containing 30 μg/mL of metconazole.

### Characterization of metconazole-resistant mutants

#### Stability of induced resistance

The metconazole-resistant mutants were studied to determine whether the resistant characteristic was stable. For this purpose, mycelial plugs from the mutants were repeatedly subcultured on fungicide-free PDA medium 10 times, and mycelial growth at the 1st and 10th subcultures was then assessed to determine the EC_50_ values. The ratio of the EC_50_ value of a resistant mutant to that of its sensitive parent was calculated to obtain the resistance factor (RF) (Duan et al. 2018), while the ratio of the RF value of the 10th generation to that of the 1st generation yielded the factor of sensitivity change (FSC) (Duan et al. 2018). Each experiment involved three replicates and was repeated three times.

#### Sensitivity to temperature

Mycelial plugs from parental and resistant strains were cultured on fungicide-free PDA for five days in the dark at 4, 13, 18, 25, 30 and 37 °C. Following the incubation process, the colony diameters were measured perpendicularly. Each experiment involved three replicates and was repeated thrice.

#### Conidia formation and germination

Three parental isolates and six resistant mutants were used to assess the production of conidia. For each isolate, five fresh fungal disks were collected from a three-day-old colony and transferred into 100 mL of 3% MBB. After a five-day incubation on a shaker at 25 °C and 180 rpm, a hemocytometer was used to determine the concentration of conidia. A conidial suspension (200 μL) was then evenly spread onto 1.5% water agar, and after incubating for 8 h in the dark at 25 °C, conidial germination was assessed under a light microscope based on 100 conidial samples. Each experiment involved three replicates was repeated three times.

#### Pathogenicity assay

The pathogenicity assay was performed as described before (Li et al. 2024a, b). Sodium hypochlorite (NaClO, 3%) was used to disinfect the surface of wheat seeds (cultivar: xiaoyan 22) for 5 min, after which the seeds were washed with sterilized distilled water three times. An incision was made approximately 1 mm above the coleoptiles of three-day-old seedlings prior to inoculation with the mycelial plugs of both the parental and mutant strains. A control was also included using a blank plug of PDA for inoculation. The inoculated coleoptiles were incubated in plastic boxes (34 × 22.5 × 10 cm) at 25 °C and under 95% humidity. After five days, the coleoptiles were analyzed to determine the length of infection. The experiment was carried out three times, with 18 replicates for each strain.

#### Efficacy of metconazole

Wheat coleoptiles were prepared as described above before being sprayed with 5 μg/mL of metconazole. For the untreated control, spraying was carried out using sterile water that contained a similar concentration of DMSO. The coleoptiles were then incubated for 24 h at 25 °C, after which mycelial plugs were collected from the mutants and their respective parental isolates and carefully positioned at the wheat coleoptiles. After a five-day incubation at 25 °C, the coleoptiles were observed to determine the length of lesions, with the following equation eventually used to calculate control efficacy:

$$\text{Control efficacy }(\%) = 1 - \frac{\text{lesion length after fungicide treatment}}{\text{lesion length of control}} \times 100$$

Three experiments were conducted, each involving eight replicates per strain.

#### Cross-resistance

The sensitivity of 26 wild-type *F. pseudograminearum* isolates and 6 metconazole-resistant mutants were assessed against 6 fungicides using the mycelia assay. The selected fungicides were pyraclostrobin, carbendazim, fludioxonil, pydiflumetofen, mefentrifluconazole and tebuconazole, with their concentrations provided in Table S2.

#### Sequencing the *FpCYP51* genes of *F. pseudograminearum*

The cetyltrimethylammonium bromide (CTAB) method was used for extracting genomic DNA from three parental strains (metconazole-sensitive) and six laboratory-generated mutants (metconazole-resistant) (Brandfass and Karlovsky 2008). PCR primers (Table S1) targeting *FpCYP51A* (FPSE_00109), *FpCYP51B* (FPSE_01496) and *FpCYP51C* (FPSE_02459) were then designed for amplifying those genes, including their upstream sequences.

For the PCR, 20-µL reaction mixtures containing 1 μL of each primer (10 μM), 10 μL of 2 × M5Taq HiFi PCR mixture (Mei5 Biotechnology Co.) and 100 ng of genomic DNA were prepared, with the amplification process subsequently performed on a T100™ thermal cycler (Bio-Rad) under the following conditions: an initial 3-min step at 94°C, followed by 35 cycles each consisting of denaturation for 30 s at 94°C, annealing for 30 s at 58°C and extension for 40 s at 72°C. The PCR was eventually completed with a final extension of 5 min at 72°C. The resulting products were then sequenced at TSINGKE Biological Technology Co., Ltd., with the results analyzed using DNAMAN version 9.0.1.

#### Plasmid construction for transformation of *F. pseudograminearum*

Gene replacement constructs were generated for the M151T point mutant in the FpCYP51B protein using the split-marker approach (You et al. 2009; Zhao et al. 2022). Following the extraction of DNA from sensitive parental strains, PCR amplification was performed with specific primers (Table S1). After ligation with a positive (the hygromycin B phosphotransferase gene, *hph*) and a negative selectable marker (the herpes simplex virus thymidine kinase gene, *hsv-tk*), sequence verification was performed prior to protoplast preparation and transformation experiments as described before (Shao et al. 2022). Suspected transformants were then plated on PDA to which hygromycin B had been added (final concentration of 100 μg/mL, Sigma-Aldrich). The M151T transformants exhibited normal growth on plates containing hygromycin B. After screening the plates, PCR was used to confirm successful transformations.

#### Sensitivity of transformants to metconazole

The sensitivity of the wild-type strain HB19 (control) and the transformants harboring the FpCYP51B-M151T mutation to 0, 0.005, 0.01, 0.05, 0.1, 0.25 and 0.5 μg/mL of metconazole was determined. This involved incubating the fungi for 5 days in the dark at 25 °C before measuring colony diameters to determine the EC_50_ values. For each concentration, the experiment was repeated three times.

#### Homology modeling and molecular docking

For this study, a well-modeled FpCYP51B structure, constructed using the AfCYP51B template (6CR2) (Friggeri et al. 2018), was applied through the web-based server YASARA (Chemical Computing Group). Sequences of the CYP51B protein were subsequently aligned, with the results revealing a 58.30% identity between *F. pseudograminearum* and *Aspergillus fumigatus* (Fig. S1). Once the model was generated, Amber 14: EHT force field was used to assess and analyze the 3D conformation of metconazole (https://pubchem.ncbi.nlm.nih.gov/) (Maier et al. 2015). The YASARA software was also used for docking experiments prior to further optimization with the MOPAC program. Through the docking analysis, differences in inhibitor binding between the wild type and mutant FpCYP51B were compared. Finally, binding affinities were evaluated based on energy scores and binding configurations.

#### Expression level of *FpCYP51s* by quantitative real-time PCR

Parental strains and metconazole-resistant mutants were grown in 100 mL of PDB for 2 days at 25 °C and with continuous shaking at 90 rpm. They were then exposed to metconazole (at the EC_50_ for each isolate) for 24 h before collecting mycelia by vacuum filtration. A control was also set up by replacing the fungicide with an equivalent volume of DMSO. A Fungal total RNA isolation kit (Genenode Biotech Co., Ltd.) was then used for extracting total RNA from the mycelia, and this was followed by the synthesis of corresponding cDNA strands, performed with One-Step gDNA Removal and cDNA Synthesis SuperMix (TransGen Biotech Co., Ltd.) as required by the manufacturer. The expression of the *FpCYP51* genes was then assessed on a CFX Connect Real-Time system (Bio-Rad) using the primers listed in Table S1 as well as the SYBR Premix kit (Tiangen Biotech Co., Ltd.). The relative expression of the genes was eventually determined by the 2^−ΔΔCt^ method against *FpTEF1α* as the reference (Wei et al. 2023; Li et al. 2023c). The above experiments were repeated thrice.

#### Constructing transformants for overexpression of the *FpCYP51A/B* gene

The cDNA of the sensitive parental isolates was amplified by polymerase chain reaction (PCR) using the primer pairs listed in Table S1. The resulting PCR product was purified using a gel extraction kit (TransGen Biotech Co., Ltd., Beijing, China), and then it was ligated into the pKNT-RP27-GFP vector as an *Xho*I/*Bam*HI fragment. After being verified by DNA sequencing, these plasmids were introduced into the protoplast of the sensitive *F. pseudograminearum* strain HB19. The preparation of protoplasts and the PEG-mediated transformation were carried out according to a previous study (Shao et al. 2022). For the transformants recovered from the culture plates containing 50 μg/mL G418, their genomic DNA and cDNA were amplified by PCR using the primers in Table S1 to confirm the presence and expression of the transgene, respectively.

#### Statistical analyses

The Data Processing System software (version 7.05) was used for analyzing all results. Specifically, EC_50_ values as well as biological characteristics, such as disease severity, CFI, conidiation, conidial germination rate and colony diameter, were compared with one-way ANOVA, followed by Tukey’s honest significant difference (HSD) test, in order to determine standard errors and assess the significance of differences at 5% significance level.

## Supplementary Information

Supplementary Material 1. Table S1 Target genes, and the sequence of primers used in this study. Table S2 Concentrations used to determine the sensitivity of wild-type isolates and metconazole-resistant mutants of *Fusarium pseudograminearum* to various fungicides. Fig. S1 Alignment of FpCYP51B with the template 6CR2.pdb. Fig. S2 Sanger sequencing traces of mutation regions of metconazole-resistant *Fusarium pseudograminearum* mutants obtained by fungicide adaption. Fig. S3 Ramachandran plot (A) and ERRAT (B) rationality evaluation of FpCYP51B protein model.

## Data Availability

Data and materials will be made available on request.

## Abbreviations

DMI: 14α-Demethylation inhibitor
EC_50_: Median effective concentration
FCR: Fusarium crown rot
FS: Flowable concentrate for seed treatment
FSC: Flowable concentrate for seed coating
SC: Suspension concentrate
FRAC: Fungicide resistance action committee
FpCYP51: *Fusarium pseudograminearum* Cytochrome P450 51
CFI: Compound fitness index
PDA: Potato dextrose agar
PDB: Potato dextrose broth
YBA: Yeast peptone acetate
MBB: Mung bean broth
CMC: Carboxymethyl cellulose
DMSO: Dimethyl sulfoxide
RF: Resistance factor
FSC: Factor of sensitivity change
