# Supplementary material for: Resistance risk asssement and molecular basis of metconazole in Fusarium pseudograminearum
Source: Stress Biol. 2025 May 1;5(1):30. doi: 10.1007/s44154-025-00221-0 (PMC12043553; doi:10.1007/s44154-025-00221-0)
Supplement: Supplementary file 1 — Supplementary Material 1. Table S1 Target genes, and the sequence of primers used in this study. Table S2 Concentrations used to determine the sensitivity of wild-type isolates and metconazole-resistant mutants of Fusarium pseudograminearum to various fungicides. Fig. S1 Alignment of FpCYP51B with the template 6CR2.pdb. Fig. S2 Sanger sequencing traces of mutation regions of metconazole-resistant Fusarium pseudograminearum mutants obtained by fungicide adaption. Fig. S3 Ramachandran plot (A) and ERRAT (B) rationality evaluation of FpCYP51B protein model. [file 44154_2025_221_MOESM1_ESM.docx]

**Table S1** Target genes, and the sequence of primers used in this study.

**Table S2** Concentrations used to determine the sensitivity of wild-type isolates and metconazole-resistant mutants of *Fusarium pseudograminearum* to various fungicides.

**Fig. S1** Alignment of FpCYP51B with the template 6CR2.pdb.

**Fig. S2** Sanger sequencing traces of mutation regions of metconazole-resistant *Fusarium pseudograminearum* mutants obtained by fungicide adaption.

**Fig. S3** Ramachandran plot (A) and ERRAT (B) rationality evaluation of FpCYP51B protein model.

**Table S1**

| **Primer** | **Sequence (5’ to 3’)** | **Use** |
| --- | --- | --- |
| FpCYP51AF | AAACCCTGCTTGAATAGTTG | Amplification of the *FpCYP51A* gene |
| FpCYP51AR | TGTCGGATCTTCACGCAAAA |  |
| FpCYP51BF | TTTCTTCCTGGCGTTGACTA | Amplification of the *FpCYP51B* gene |
| FpCYP51BR | ATACCAGCCCAGCCATTACA |  |
| FpCYP51CF | ACGATGCTGACCGAACTTGC | Amplification of the *FpCYP51C* gene |
| FpCYP51CR | CTGGCGAAATCATGGGTTGA |  |
| SFpCYP51AF | TTGAGCTCAATTAGGATGGT | Amplification of the partial upstream regions of *FpCYP51A* gene |
| SFpCYP51AR | GTGAAAAACTAGCGGAGGTT |  |
| SFpCYP51BF | TCGGACCATGCCATAGCCCT | Amplification of the partial upstream regions of *FpCYP51B* gene |
| SFpCYP51BR | GCTCATTGGGGTTTCGGAAG |  |
| SFpCYP51CF | TTGGGTATACGGGATTGAAG | Amplification of the partial upstream regions of *FpCYP51C* gene |
| SFpCYP51CR | GCATCGTTCGTCTCGAGGTT |  |
| qFpCYP51AF | ACGCCGAAGAAGTTTACGGT | qRT-PCR of *FpCYP51A* expression |
| qFpCYP51AR | CTCAGCCATTGCCTTGGAGA |  |
| qFpCYP51BF | TGCAAACCATTGTTGCCGAG | qRT-PCR of *FpCYP51B* expression |
| qFpCYP51BR | GAGAAGAGCGAGGCGTAGTC |  |
| qFpCYP51CF | TTCGTCATGCCAGGTCTTCC | qRT-PCR of *FpCYP51C* expression |
| qFpCYP51CR | AACCATATCCGTCTCGTCGC |  |
| FpTEF1a-RTF | TCACCACTGAAGTCAAGTCC | Internal standard gene for qRT-PCR |
| FpTEF1a-RTR | ACCAGCGACGTTACCACGTC |  |
| Fp1-1 | CGGGGTAGTTTCACATTTC(C/T)G | Identification of *F. pseudograminearum* isolates |
| Fp1-2 | GAGAATGTGATGA(C/G)GACAATA |  |
| BF1 | AGCAGTTGCTAGTGAATCTGTGAT | Amplify *FpCYP51B*-M151T homologous upstream region |
| B151R | GGCTTCGGTGGTCAGGGCAATCTTCGTGAAC  TAGGAGTGTATGTCAGTATAC |  |
| B151F | GTATACTGACATACACTCCTAGTTCACGAA  GATTGCCCTGACCACCGAAGCC | Amplify *FpCYP51B* -M151T homologous downstream region |
| R | AATATCAGTTGGCAAGCTGCTCTAGTTACTGGCGTCGCTCCCAGTGAATG |  |
| HygF | CATTCACTGGGAGCGACGCCAGTAACTAGAGCAGCTTGCCAACTGATATT | Amplify the two selectable markers |
| HygR | TCATTGCCGTCAGGCATAAGTGTCGACTTAATAACACATTGC |  |
| XF | CGCAATGTGTTATTAAGTCGACACTTATGCCTGACGGCAATGA | Amplify the partial DNA sequence of the *FpCYP51B* gene |
| XR | ACAAACTCATCCCAATCTG |  |
| YZ-BF | CATAGCCCTGAAAGCTTTTC | Amplify upstream-HPH fragment |
| HY-R | GTATTGACCGATTCCTTGCGGTCCGAA |  |
| YG-F | GATGTAGGAGGGCGTGGATATGTCCT | Amplify HPH-downstream fragment |
| YZ-BR | AGCAGCTGTGGCTTGGAGAACTGG |  |
| OEFpCYP51AF | AATCTTCAAACTCGAGATGTTCCATCTACTCATCTATCCC | Amplification of the *FpCYP51A* gene ligated to vectoer |
| OEFpCYP51AR | AACGTTAAGTGGATCCCTATGTCTTCTTCCTACGCTCCC |  |
| OEFpCYP51BF | AATCTTCAAACTCGAGATGGGTCTCCTTCAAGAACTGGC | Amplification of the *FpCYP51B* gene ligated to vectoer |
| OEFpCYP51BR | AACGTTAAGTGGATCCTTACTGGCGTCGCTCCCA |  |
| PKNTG-F | CTGATTGATATCTCGTGTTCTCT | Universal primers of expression vector |
| PKNTG-R | AGATCCTGAACACCATTTG |  |

**Table S2**

| **Fungicide** | **Culture medium** | **Concentration (µg/mL)** | |
| --- | --- | --- | --- |
|  |  | **metconazole-sensitive isolates** | **metconazole-resistant mutants** |
| Tebuconazole | PDA | 0, 0.025, 0.05, 0.1, 0.4, 0.8, 1 | 0, 0.1, 0.4, 0.8, 1, 2.5, 5 |
| Mefentrifluconazole | PDA | 0, 0.01, 0.05, 0.1, 1, 5, 10 | 0, 0.05, 0.1, 1, 5, 10, 20 |
| Pydiflumetofen | YBA | 0, 0.005, 0.01, 0.03, 0.06, 0.1, 0.5, 1 | 0, 0.005, 0.01, 0.03, 0.06, 0.1, 0.5, 1 |
| Pyraclostrobin | PDA | 0, 0.01, 0.05, 0.1, 0.5, 1, 5, 10 | 0, 0.01, 0.05, 0.1, 0.5, 1, 5, 10 |
| Fludioxonil | PDA | 0, 0.01, 0.03, 0.06, 0.1, 0.5 | 0, 0.01, 0.03, 0.06, 0.1, 0.5 |
| Carbendazim | PDA | 0, 0.2, 0.4, 0.6, 0.8, 1, 2 | 0, 0.2, 0.4, 0.6, 0.8, 1, 2 |

**Fig. S1**

**Fig. S2**


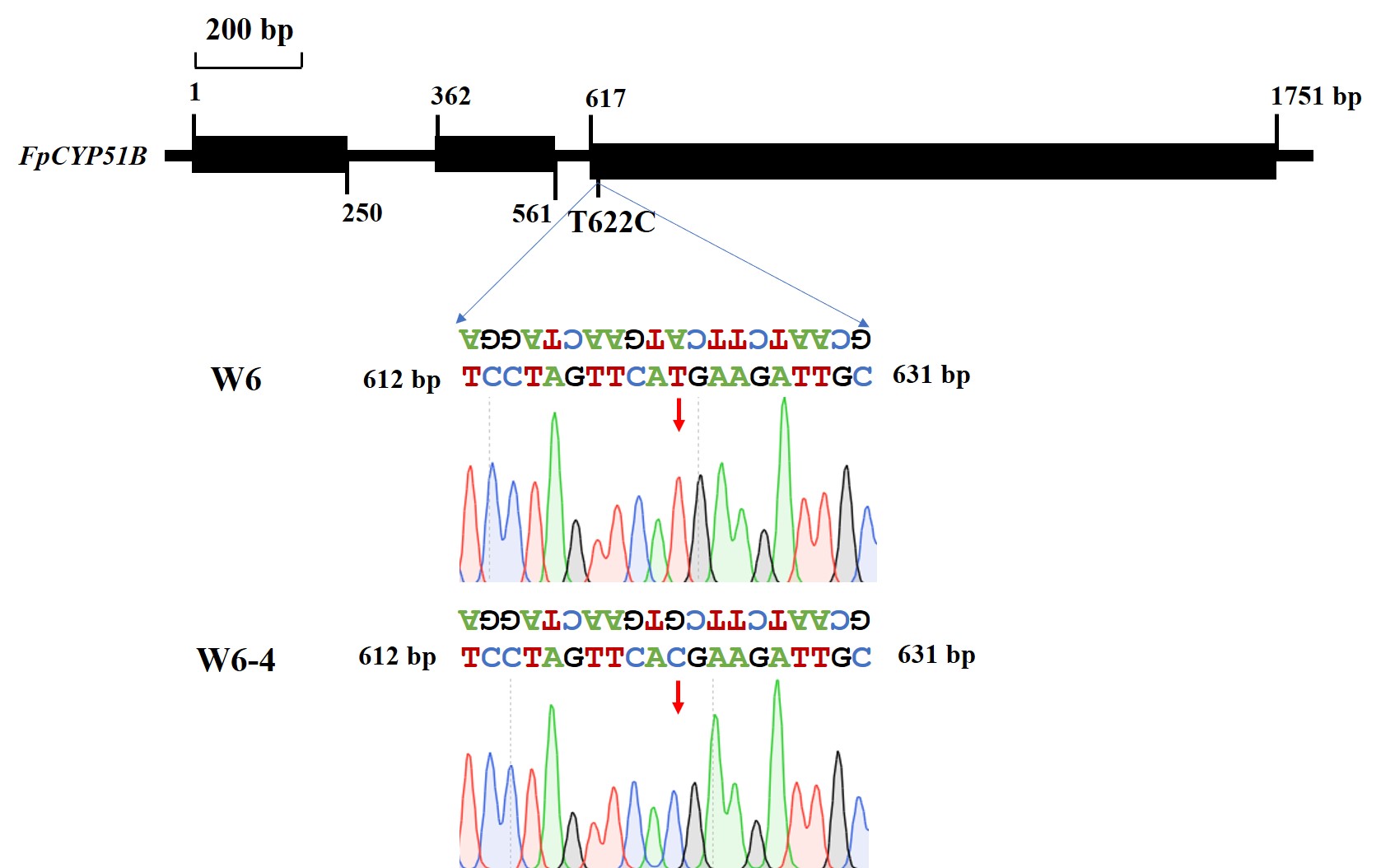


**Fig. S3**

**
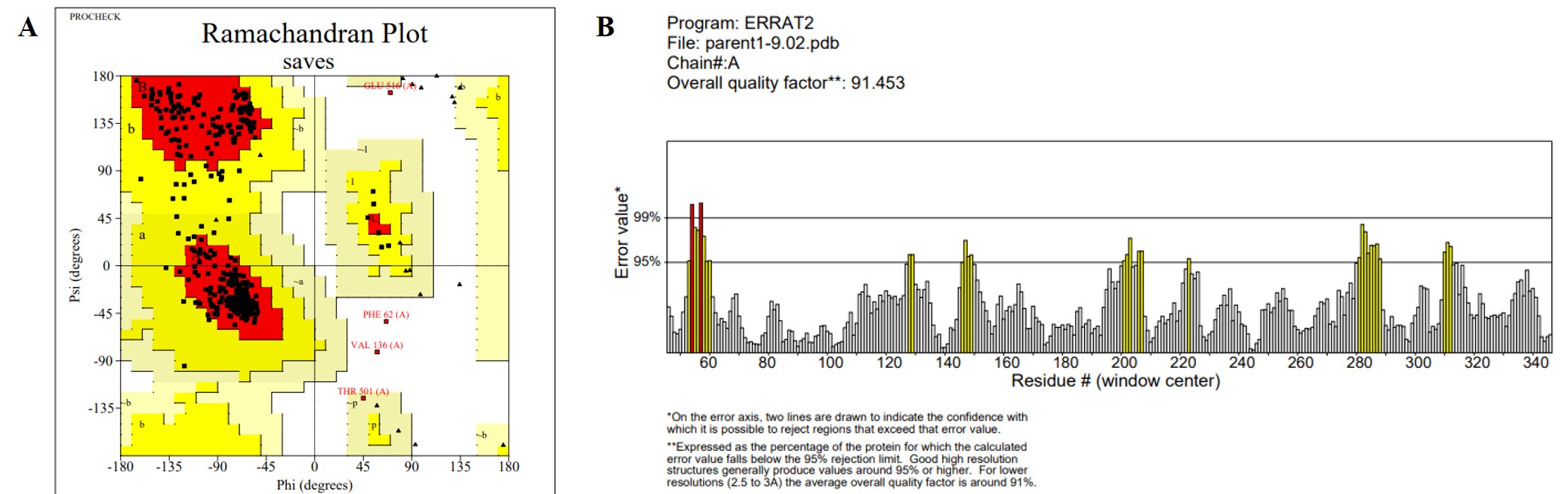
**
